# Understanding users of online energy efficiency counseling: comparison to representative samples in Norway

**DOI:** 10.3389/fpsyg.2024.1364980

**Published:** 2024-08-06

**Authors:** Christian A. Klöckner, Alim Nayum, Stepan Vesely

**Affiliations:** ^1^Department of Psychology, Norwegian University of Science and Technology, Trondheim, Norway; ^2^Department of Psychology, University of Bergen, Bergen, Norway

**Keywords:** energy efficiency, renovation, one-stop-shops, counseling, psychological drivers, theory of planned behaviour, personal norms, facilitators

## Abstract

**Introduction:**

To achieve substantial energy efficiency improvements in the privately owned building stock, it is important to communicate with potential renovators at the right point in time and provide them with targeted information to strengthen their renovation ambitions. The European Union recommends using one-stop-shops (OSSs), which provide information and support throughout the whole process, from planning to acquisition of funding, implementation, and evaluation as a measure to remove unnecessary barriers.

**Methods:**

For this paper, we invited visitors of two Norwegian websites with OSS characteristics to answer an online survey about their renovation plans and energy efficiency ambitions. The participants visited the websites out of their own interest; no recruitment for the websites was conducted as part of the study (*N* = 437). They also rated a range of psychological drivers, facilitators, and barriers to including energy upgrades in a renovation project. Their answers were then compared to existing data from representative samples of Norwegian households regarding home renovation in 2014, 2018, and 2023, as well as data from a sample of people who were engaged in renovation projects in 2014, which was collected by the research team with a similar online survey. Furthermore, 78 visitors completed a brief follow-up online survey one year later to report the implemented measures.

**Results:**

We found that visitors of the websites are involved in more comprehensive renovation projects and have substantially higher ambitions for the upgrade of energy efficiency compared to the representative samples. They also perceive stronger personal and social norms, as well as have a different profile of facilitators and barriers.

**Discussion:**

The findings suggest to policymakers that OSSs should be marketed especially to people motivated to upgrade energy efficiency but lack information and are unable to implement their plans alone. Also, the construction industry might refer interested people to such low-threshold online solutions to assist informed and more ambitious decisions.

## 1 Introduction

Reducing energy use in the building sector by increasing energy efficiency is a key pillar of decarbonising Europe as formulated in the EU’s “Fit for 55” legislation (Schlacke et al., 2022, 4). On a global level, the residential sector is the third largest energy consumer, representing 27–30% of the energy consumption, almost at the same level as transportation and industry (Nejat et al., 2015, 843; IEA, 2023). Also in Europe, the residential sector stands for 26% of final energy consumption, being the second largest consumption sector after transportation (Tsemekidi et al., 2019, 1). Whereas the primary energy consumption in the residential sector decreased by 4.6% between 2000 and 2016 (Tsemekidi et al., 2019, 9), there is still a substantial untapped potential for further improvement of energy efficiency in the sector. This can be achieved through energy efficiency renovation of the existing building stock (Pohoryles et al., 2020, 11–12). Realizing this potential requires that also private house owners invest in energy efficiency measures. However, the annual rate of housing renovation in Europe is only about 1% (Biere-Arenas and Marmolejo-Duarte, 2022, 185), which is far too slow to reach the ambitious energy conservation targets. Besides, not all of those renovations include energy efficiency improvements. This raises the question of how property owners make decisions about renovating and energy efficiency measures and how they can be efficiently supported in these processes. To alleviate this problem, one-stop-shops (OSS), which are places where interested citizens can get counseling and support for the whole process of an energy retrofit, have gained a lot of attention lately as a means to support citizens in the matter of energy retrofits also from the European Union (as for example reflected in recently finished EU projects like “EUROPE one stop” or “ProRetro”).

### 1.1 One-stop-shops in energy counseling

Bertoldi et al. (2021, 3–12) analysed the role of OSSs across Europe. They concluded that OSSs may be able to address some of the main barriers that households face when deciding about energy efficiency renovations. Often, these barriers can be categorized as economic (upfront costs, need for loan, split incentives between landlords and renters/disagreement between owners), information (information asymmetries, outcome uncertainties, incorrect beliefs), and decision-making (limited attention, social invisibility of the action, cognitive burden, loss aversion, status quo bias). Their analysis of 63 OSSs over Europe showed that the services the OSSs offer differ considerably, as do their business models. Some of them are public entities that often offer services for free, others are commercial enterprises. Their clients are usually homeowners living in relatively old buildings, and only a few of them work with social housing. Also Bagaini et al. (2022, 3–4) analysed and categorized 29 OSS initiative around Europe and formulated five key elements on which the different OSS differed: (a) value proposition, (b) services, (c) partnership management, (d) revenue stream, and (e) shared value. Based on these dimensions, they destilled three archetypes for OSS models: They refer to them as the Facilitation Model (mostly focused on providing information to homeowners without a revenue generation model behind), the Coordination Model (also taking in a project management role with the contractors and generating revenue by fixed fees), and the Development Model (similar to the Coordination Model but with a revenue generated dynamically from the shared energy savings). Along similar lines, Pardalis et al. (2022) compared publicly and privately funded OSSs. In addition to the facilitation and the coordination model they separate the development model into “all inclusive models” (where the renovation process is fully managed by the OSS under one single contract, but energy savings are not guaranteed) and “ESCO models” (where Energy Service Companies−ESCOs−manage the whole renovation package and also guarantee energy savings). Whereas publicly funded OSSs are evaluated as providing homeowners with crucial services at the right time, privately funded OSSs struggle more with generating revenue and providing access to financing.

According to Bertoldi et al. (2021), a key activity all of the surveyed OSSs cover is the assessment of the status quo, which is done in different ways (sometimes as a guided online self-assessment). Then, a stage of guidance toward possible measures is started, usually resulting in an individual renovation plan. In the next stage, financing is secured (either directly or indirectly, for example by supporting applications for subsidies). In the implementation stage, OSSs either manage the implementation themselves or recommend contractors who will do that. Often OSSs are involved in quality assurance of the implemented measures afterwards, sometimes certifying the result. Some OSSs also monitor the building after the energy upgrade to support the clients, often through a contract where financial benefits are shared between the OSS and the client (often in ESCO models). Finally, most OSSs also engage in campaigns for energy efficiency in buildings to increase awareness.

McGinley et al. (2020, 355–57) formulate some key considerations for OSS design. They define OSS as offering full-service retrofitting, including initial building evaluation and thorough analysis, proposal of retrofitting solutions, retrofit execution, and quality assurance. However, they also state that little is known about characteristics and motivations of households that are drawn to OSS and how household decisions are impacted by OSSs, a research gap we aim to fill with this paper.

A number of recent EU projects have addressed the issue of OSSs in detail. In particular, the “EUROPA one stop” project (europaonestop.eu) is interesting as it created an online platform (SUNShINE−savehomesave.eu) to connect homeowners, facility managers, and contractors working on energy efficiency upgrades and provide them with easy access tools to online diagnose their renovation potential. This platform is structurally comparable with the platforms analysed in this paper and can be considered a concept following the facilitation model. However, to understand how homeowners may be affected by OSSs, it is important to take a look at decision-making processes.

### 1.2 Psychological drivers of implementing energy efficiency in renovation of privately owned dwellings

In a detailed study of decision-making about energy retrofits in Norwegian households data of which was also used as a comparison for this study, Klöckner and Nayum (2017, 1014) found that an extended Theory of Planned Behaviour (Ajzen, 1991, 182; Klöckner, 2013, 1032) formed a viable theoretical framework to structure these decision processes. They were able to show that personal norms, positive attitudes, and high self-efficacy were the decisive factors for forming intentions to include energy efficiency upgrades in renovation projects. Social norms were closely related to personal norms and an important trigger of these. More distal factors were problem awareness, value orientations, perceived consumer effectiveness, and innovativeness. The most central concepts are briefly introduced in the next paragraph.

In this context, personal norms are a feeling of moral obligation to invest in better energy efficiency. Positive attitudes are the overall evaluation of the pros and cons of the decision to invest. That is how good or bad this would be, all taken into account. Self-efficacy captures how capable one feels to implement the investment, a factor that most likely will be directly affected by engaging with an OSS. Following the theoretical framework as outlined and tested by Klöckner and Nayum (2017, 1014), an intention to invest will thus be formed: (a) if people feel that they are morally obliged to do that because wasting energy is a bad thing which is more likely; (b) if other people who are important to them support this view. Furthermore, c) a positive attitude to energy efficiency investments d) and a high self-efficacy (i.e., knowing how to implement these measures and/or who to contract to do it) also contribute. As attitudes are a combination of positive and negative beliefs about the behavioral alternatives that people choose between (Ajzen, 1996, 385–403), a closer look at assumed barriers and facilitators underlying those alternatives could help in understanding the decision process further, as discussed in the next section.

### 1.3 Barriers and facilitators of energy efficiency measures in buildings

A number of studies analyzed facilitators of or barriers against implementing energy efficiency in a residential building from different theoretical and methodological perspectives. In his PhD thesis, Pardalis (2021, 60) finds, based on an online survey with almost 1000 homeowners in Sweden, that the house age and time lived in a house but also energy concern trigger the decision to renovate. These factors are, again, influenced by sociodemographic factors of the occupants. Thus, structural aspects seem of importance as drivers of the retrofit decision.

Digging deeper into the decision process, Xue et al. (2022, 5) conducted interviews with 39 professionals in the retrofit market to identify barriers to energy retrofitting from the perspective of the public sector, the private sector, and the owners who conduct the retrofit. They found financial issues as the most important barrier in all three groups. For owners who are supposed to implement energy efficiency measures, they further named lack of information, lack of creative models or cases, risks connected to the project, trust, and negative social influence as important barriers. Also, problems of reaching an agreement, time consuming processes, limited added value, and concerns about payback time were named.

Many of these aspects were also reflected in another qualitative study. Klöckner et al. (2013, 406–408) interviewed 70 Norwegians on drivers and barriers regarding energy efficiency behaviour. They found that economic barriers (e.g., lack of investment money), motivational barriers (e.g., too much effort, loss of comfort, low perceived efficacy), structural barriers (e.g., building structure, ownership), and informational barriers (e.g., lack of trust, uncertainty, lack of specific information) were central.

Departing from practice theory in an ethnographic study of renovation projects, Judson and Maller (2014) interviewed 49 Australians involved in renovation projects and unraveled the process of renovation even more. They found that renovation projects, to a large degree, are shaped and reshaped by the existing or evolving practices people have within their buildings. Energy efficiency is traded off against other needs and meanings, negotiation between different household members occur, and focus shifts dynamically. Some parts of the home have a meaning for its inhabitants as part of their daily practices which cannot just be changed to enhance energy efficiency.

With a quantitative perspective, Klöckner and Nayum (2016, 5) studied barriers in different stages of renovation processes in a representative sample of Norwegian households. Their findings indicate that facilitators like perceived increase in comfort, anticipated better living conditions or increased marked value were important in the early stages of decision making. Information about subsidy schemes or trustworthy information about the procedures came out as important at a later stage when planning was more advanced. Correspondingly, some barriers like building protection regulations, planning to move soon, or not owning the building were relevant already early in the process before people started even thinking about an energy retrofit, whereas barriers like too much disturbance of everyday life, contractors with a lack of competence, the need to supervise contractors, or a lack of economic resources were turned out to be relevant barriers later in the process. A particularly important barrier appeared to be the feeling that “the right point in time for a larger renovation project has not come, yet”.

In an economic modeling approach comparing expected utility theory (which assumes that decision makers chose the alternative with the best possible utility for them) and cumulative prospect theory (which assumes that decisions about investments are strongly affected by specific decision biases), Ebrahimigharehbaghi et al. (2022) found that cumulative prospect theory, which takes biases like “reference dependence” (utility changes are interpreted differently with respect to difference reference points), “loss aversion” (losses weigh higher than gains of the same size), “diminishing sensitivity” (avoiding risk for positive outcomes but taking risks for negative outcomes), and “probability weighting” (events with low probability but more extreme outcomes are overestimated) is much better equipped to predict homeowners investments in home energy efficiency in a large sample from the Netherlands than classical expected utility theory. This shows that people’s decision-making in such cases takes other aspects than economic utility into consideration to a large degree.

Studies such as the ones briefly mentioned above show that the selection of aspects that can interfere with or facilitate a decision-making process about energy retrofits is plentiful. In addition, they even have different importance depending on where in the process a decision-maker is. This makes it demanding to provide the most helpful support for decision-makers in the residential sector. It seems important to provide the right information at the right time to the right people, which underscores the need for careful targeting and timing of information provision. Flexible and interactive online counseling systems, which can take people through all stages of the process, similar OSSs, may be a way to find a good balance between resources needed and effects achieved in targeted energy counseling. Interestingly, Pardalis (2021, 66) asked homeowners what would be most important for them with respect to OSSs, and guarantees for costs and quality, as well as having one contact and one contract and a preliminary check and counseling were on top of the list, directly addressing some of the issues identified as barriers in many of the studies above.

### 1.4 The present study

Summarizing what has been outlined in the introduction, energy efficiency upgrades of residential buildings are a major contributor to reaching the targets of the energy transition of the European Union. However, the private residential sector is lagging behind in this process. Renovation rates of the aging building stock are low. Even when the buildings are renovated, energy efficiency measures are not always implemented. In cases where some energy efficiency measures are included, they are often not to the standard that would be recommendable. One-stop-shops have been heavily promoted recently as a way of removing the burden of planning, financing, and implementing a deep renovation project from the individual house owners. Consequently, many such services have been implemented around Europe with differing business models, financing, and mandate. However, relatively little is known about who uses these services and what effect they have on their users. Especially, it is unknown to a large degree how interacting with a low-threshold digital OSS following a facilitation model shapes its users’ perception of barriers and facilitators of a retrofit decision, and if it affects their motivations and ambitions for this project. This research gap is addressed by the present study. More specifically, we are analysing if visitors of energy efficiency counceling websites differ in their engagement in retrofits, their energy efficiency ambitions, the profile of psychological variables, the drivers and barriers from representative samples of the population and a sample of home renovators.

Our study is, thus, contributing to the literature by providing new insights into how natural users of websites with OSS characteristics differ from the general population of homeowners on a number of psychological and socio-demographic characteristics. This helps on the one hand to identify who are the target group for such low-threshold website services, but on the other hand, we also provide an assessment if their renovation ambitions, and especially the level to which they intend to implement energy efficiency measures in these updates differs after they visited the service. Through a one-year follow-up, we can also provide an assessment of to which degree the planned measures were implemented. Taken together, the focus on primarily psychological drivers and barriers of energy efficiency investments in homes for a very specific target group in comparison to large, representative samples of homeowners paints a new, and informative picture of who the users of these websites are not only socio-demographically, but also psychologically, what they are looking for on these websites, and to which degree the websites support them in their pathway towards more energy efficient homes. Being able to run the comparisons of a relatively large sample of website users to several, large representative comparison samples which were surveyed with the same methodology in the same country over the course of 10 years provides an unique opportunity to understand the target group.

## 2 Materials and Methods

### 2.1 Study design

For this study, we collected responses from users of two online energy efficiency counseling websites, which have a similar structure that might be conceptualized as OSS following a facilitating model. These websites offer an analysis of the current energy standard of privately owned residential buildings (either as a guided self-assessment or based on data from the Norwegian building registry). They can also suggest a rough renovation plan and connect the homeowner to potential contractors who can implement energy efficiency measures. Moreover, they can provide information about costs, pay-off rates, subsidies (incl. information on how to apply), etc. Energismart.no is promoted by the environmental organization Friends of the Earth Norway, whereas energiportalen.no is promoted by Viken county. From January 2022 until January 2023, participants for the study were recruited from natural visitors of both websites by messages on the websites and pop-up windows, which promoted participation in our study and provided a link to the online questionnaire. We thus recruited people who visited the websites out of their own interest without promoting using the websites from our end. This sampling strategy was chosen to recruit a ecologically valid group of website users.

In the online survey, participants were then asked about their plans for retrofitting their homes, recently finished or ongoing retrofitting projects, the ambitions for energy efficiency upgrades as part of these retrofits, and psychological drivers and barriers of the decisions.

Since randomization of users of the websites was not possible, as people self-assigned to the websites, we chose a comparison group design, where we compared the means and distributions of key variables in our survey against representative homeowner data collected in 2014, 2018, and 2023 (Klöckner and Nayum, 2016, 2017; Egner and Klöckner, 2021; Egner et al., 2021; Peng and Klöckner, 2024) with the same survey instrument (see Table 1 for an overview of the survey samples). Because of that design, we are unable to draw causal conclusions, but we can get indications for differences between the samples (for a deeper discussion, see the limitations section below). We were also not able to survey our participants before they entered the websites. Thus, we do not know if the described differences were already there before they used the website, or which differences were caused by the website visit. It is likely that people visit such counseling websites when they already have developed an interest for the information presented there. Thus, some of the differences will have existed already pre-visit. Especially some of the drivers and barriers, but also some parts of the psychological profile might fall into that category and it is important to keep this in mind when interpreting the results. Furthermore, we do not know how long people stayed on the websites, what they read, and how much they used the information to adapt their renovation strategy, which would have given us more insights into their user experience. However, we believe that comparing the visitors to representative homeowners from different historical points in time in the same country surveyed with the same questionnaire can give us some relevant insights and at least input for generating new hypotheses.

**TABLE 1:** Overview of sample statistics in the different samples.

	Energy efficiency counseling website users	One year follow up website users	Representative sample of Norwegian households	Representative sample of Norwegian households	Representative sample of Norwegian households	Sample of Norwegian households in a renovation project
Year of data collection	2022–2023	2023	2014	2018	2023	2014
Source	Collected for this study	Collected for this study	Originally collected for Enova	Originally collected for Enova	Originally collected for the BEHAVIOR project	Originally collected for Enova
Number of participants	437	78	2,605	3,807	1,314	1,182
**Gender**						
Female	231 (52.9%)	27 (34.6%)	1,332 (51.1%)	1,858 (48.8%)	657 (50.0%)	543 (45.9%)
Male	199 (45.5%)	51 (65.4%)	1,273 (48.9%)	1,949 (51.2%)	656 (50.0%)	639 (54.1%)
Other	1 (0.2%)	0 (0%)	0 (0%)	0 (0%)	0 (0%)	0 (0%)
Mean age (SD)	53.0 (12.6)	54.4 (13.5)	49.2 (15.7)	52.7 (16.5)	48.5 (17.2)	50.6 (15.1)
**Highest education**						
Basic education	14 (3.2%)	1 (1.3%)	214 (8.2%)	191 (5.0%)	60 (4.6%)	63 (5.3%)
Vocational school	48 (11.0%)	12 (15.4%)	977 (37.5%)	955 (25.1%)	321 (24.4%)	268 (22.7%)
College	34 (7.8%)	5 (6.4%)	658 (25.3%)	352 (9.2%)	149 (11.3%)	220 (18.6%)
University	339 (77.9%)	60 (76.9%)	756 (29.0%)	2,309 (60.6%)	783 (59.6%)	631 (53.4%)
**Median gross household income**						
NOK	800,000–999,999	1,000,000–1,199,999	600,000–799,000	800,000–999,999	Individual gross income: 500,000– 599,999	800,000–999,999
EURO	70,400–88,000	88,000−105,600	52,800–70,400	70,400–88,000	44,000–52,800	70,400–88,000
**Type of house**						
Detached house	293 (67.2%)	56 (71.8%)	1,436 (55.1%)	1,997 (52.7%)	625 (47.6%)	765 (64.7%)
Duplex	51 (11.7%)	13 (16.7%)	163 (6.3%)	252 (6.6%)	83 (6.3%)	88 (7.4%)
Terraced house	45 (10.3%)	5 (6.4%)	342 (13.1%)	480 (12.7%)	140 (10.7%)	136 (11.5%)
Apartment building	35 (8.0%)	2 (2.6%)	505 (19.4%)	869 (22.9%)	367 (27.9%)	157 (13.3%)
other	12 (2.8%)	2 (2.6%)	159 (6.1%)	194 (5.1%)	98 (7.4%)	36 (3.1%)
**Ownership**						
Owning	418 (96.6%)	75 (96.2%)	2,198 (84.4%)	3,344 (88.1%)	1,113 (84.7%)	1,100 (93.0%)
Renting	15 (3.5%)	3 (3.8%)	407 (15.6%)	451 (11.9%)	194 (14.8%)	82 (6.9%)

Differences between the samples were identified by comparing 95% confidence intervals for the means. Non-overlapping confidence intervals were interpreted as significant mean differences. Effect sizes for the differences are presented in Supplementary Appendix Table 1.

One year after the participants answered the survey, we approached them again with a short survey asking if and which retrofitting measures had been implemented in the meantime and if not, why. The follow-up survey was sent to every participant who agreed to be contacted again.

### 2.2 Survey

The surveys conducted in all different studies compared here were collected through an online survey platform operated by the University of Oslo (Nettskjema.no). The questions used for the analyses presented in this paper composed only part of the questionnaires; we describe only the relevant questions below. The full survey can be found in the data repository together with the dataset.^1^

#### 2.2.1 Sociodemographic information

In the surveys, participants were asked about their gender, age, highest education level, gross household income (in the 2023 data collection, individual gross income was recorded), the type of house they lived in, and if they owned or rented etheir dwellings. The categories of these variables can be found in Table 1.

#### 2.2.2 Deep renovation

To capture if the participants were just finished, engaged in, or planning what we refer to as a “deep renovation” project, we asked them the following questions:

(1) Within the previous three years, were you involved in a renovation project that involved (a) substantial work on the roof like replacing all tiles, (b) replacing at least 50% of the outer walls, (c) replacing at least 50% of the window area, and/or (d) substantial work on the foundation? This definition was developed for the 2014 study in a collaboration of the researchers behind the studies and the Norwegian Energy Efficiency Agency Enova and used in the same form in all data collections since. The aim of this definition was to differentiate larger renovation projects from smaller, more cosmetic renovation projects.
(2) Are you currently involved in a renovation project according to the definition above or are you planning to engage in such a renovation project within the next three years?

However, the definition does not automatically assume that energy efficiency measures are included in the deep renovation project.

The ambition level of these renovation projects was measured by how many of the four components they (are planning to) implement, and it ranges from 1 to 4.

#### 2.2.3 Energy efficiency upgrade

If participants answered “yes” to either or both of the questions presented in the previous section, they were asked if that renovation project included, includes or is planned to include (a) additional insulation of the roof of at least 10 cm, (b) adding additional insulation to the walls of at least 5 cm, (c) energy saving windows with a μ-value of 1.0 or lower, (d) at least 5 cm additional insulation to the foundation walls, (e) installation of mechanical ventilation, and/or (f) installation of balanced ventilation. Also here, the definition of these measures was agreed upon with Enova in 2014 to represent a substantial improvement in the energy standard of the respective building component. For our analyses, we counted the number of these measures that had been/were planned to be implemented in the deep renovation project. The number could thus be between 0 and 6.

#### 2.2.4 Personal norms, social norms, attitudes, and efficiency

Based on the Theory of Planned Behaviour (Ajzen, 1991, 182) extended by personal norms from the Norm-Activation Model (Schwartz and Howard, 1981), four psychological variables are central to understand people’s intentions: attitudes, social norms, perceived behavioral control or behavioral efficacy, and personal norms. Each of these variables was measured by two items in the surveys, with a 7-point Likert scale from −3 to +3. Higher values indicate stronger norms, attitudes, or efficacy.

The two items to measure social norms were “People who influence my decisions think I should insulate my home” and “People who are important to me think I should retrofit my home”. The two items to measure perceived efficacy were “I know which person or company I need to contact to have my home professionally insulated” and “I know what I need to do to insulate my home”. The two items to measure personal norms were “Because of my values/principles, I feel obliged to insulate my home” and “I feel personally obliged to retrofit my home”. For each pair of items, the mean score was calculated and used in subsequent analyses.

Attitudes were measured with four semantic differentials: “Increasing the energy standard of my home would be (a) useless−useful, (b) uncomfortable−comfortable, (c) unfavorable−favorable, and (d) bad−good”. Each pair has −3 as the anchor for the negative word and +3 as the anchor for the positive word. For further analyses, the mean of the four items was calculated.

All items had been used in an identical way since the first study in 2014, as documented elsewhere (Klöckner and Nayum, 2016, 2017). In the 2023 data collection, different answering scales were used, therefore the results are not comparable and are not reported here (Peng and Klöckner, 2024).

#### 2.2.5 Barriers and facilitators

Finally, a list of potential barriers and facilitators of energy efficiency upgrades was presented in random order to the participants, asking how much they agreed with each item. The items can be found in the Supplementary Appendix. These lists were derived from a qualitative study on reasons why Norwegians upgrade or decide not to upgrade energy standards of their dwellings (Klöckner et al., 2013). In the 2023 data collection, different answering scales had been used, therefore the results are not comparable and are not reported here.

### 2.3 Sample and comparison groups

**FIGURE 1:**
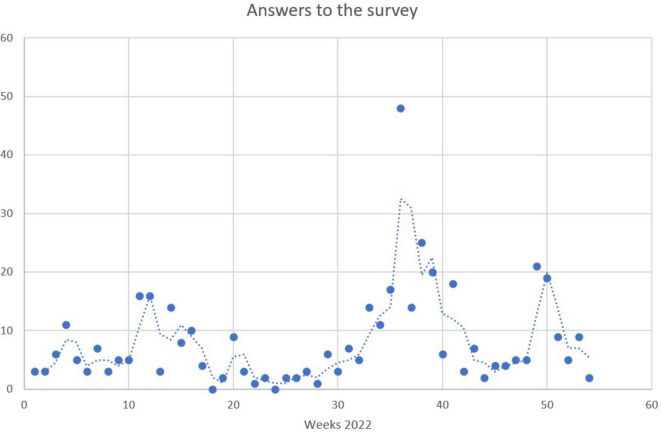
Number of participants recruited for the counseling website user survey per week in 2022 (the line is the moving average).

The sample of counseling website users was recruited from the first week of January 2022 to the first week of January 2023. In total, 437 answers were collected. These answers were not equally distributed over the year, however, as (Figure 1) shows. Whereas relatively many responses were collected in winter and early spring 2022, the interest was reduced in late spring and summer before it skyrocketed after summer 2022, as well as in winter 2023. This coincided with electricity price peaks in Norway (especially in the South) and media discussions about that topic. Thus, a first conclusion can already be that the interest in using energy efficiency counseling websites clearly follows the pattern of the energy price fluctuation and accompanying societal discussion.

Table 1 below shows the sociodemographic statistics of the sample from the counseling websites in comparison to the existing samples in detail. As can be seen, the samples are comparable on most of the dimensions. All samples contain close to 50% males and females (with the most deviation in the sample of renovators from 2014). The average age is around 50 years in all samples, with the youngest average age in the 2023 population sample and the oldest average age in the sample of the users of the websites. Education varies quite strongly, with the population sample from 2014 being the outlier with far lower education level than all other samples. Participants recruited from the counseling websites had the highest education level. The median household gross income category is the same in most samples. However, it is lower in the 2014 population sample and higher in the sample of people who answered the one-year follow-up after the visit on the counseling websites. Income categories of the 2023 sample cannot be compared, as individual gross income was recorded in that data collection. However, it can be extrapolated that the average household income would be comparable to the other samples. The proportion of people living in detached houses is particularly high in the sample of website users and the renovator sample from 2014. Also, the level of people owning their dwelling is close to 100% in these groups and a little lower in all other groups. As a conclusion, it can be said that the samples are comparable on most dimensions. Meanwhile, the website users are most similar to the people who were recruited as being in a renovation project in 2014. That is, they are more likely better educated, more likely to live in a detached house, and more likely to own their dwelling than representative samples of Norwegian households.

## 3 Results

In the following section, we present the results of the comparison of the counseling website users with the other available samples. To do this, we examine the 95% confidence intervals as displayed in the figures for overlaps between the group of website users and the other groups. As the data is partly in separate datasets, we did not calculate formal significance tests, but a non-overlapping 95% confidence interval corresponds to an assumed significant difference between the respective groups. The numbers for the website users are always highlighted in the figures. Effect sizes are reported in Supplementary Appendix Table 1. An overview of all results can be found in Table 2.

**TABLE 2:** Summary of the differences between the website visitors and the representative homeowner samples from 2014, 2018, and 2023, as well as the renovator sample from 2014.

	2014	2018	2023	Renovators (2014)
Renovation conducted	+	0	+	−
Renovation ongoing/planned	0	0	+	−−
Renovation levels conducted	+	−−	+	+
Renovation levels ongoing/planned	++	++	++	++
EE levels conducted	++	++	+	++
EE levels ongoing/planned	+++	+++	++	+++
Attitudes	+	+		0
Personal norms	+++	+++		++
Social norms	++	++		+
Self-Efficacy	−	0		−
More comfort	+	+		0
Cost reduction	++	++		+
Better life	+	++		+
Information trust	0	0		+
Increased value	+	+		0
Health effects	0	+		0
Energy waste	++	++		+
Info easy to find	−−	−−		−
Subsidy	−	−−		−
Short payback time	0	0		−
Much time	+	+		+
Lack of money	++	++		++
Disruption	+	0		+
Not right time	−	−−		0
Lacking trust	0	0		0
Info difficult to find	++	++		+
Cannot decide	++	++		+
Builders lack knowledge	++	++		++
Moving out	+	0		+
Must agree with neighbours	0	0		0
Negative experience	0	+		0
Building protection	0	+		0
Renting	0			0

+, the website users score higher with a small effect size, ++, the website users score higher with a medium effect size, +++, the website users score higher with a large effect size, −, the website users score lower with a small effect size, −−, the website users score lower with a medium effect size. For the exact effect sizes, please see Supplementary Appendix Table 1.

### 3.1 Engagement in deep renovation

**FIGURE 2:**
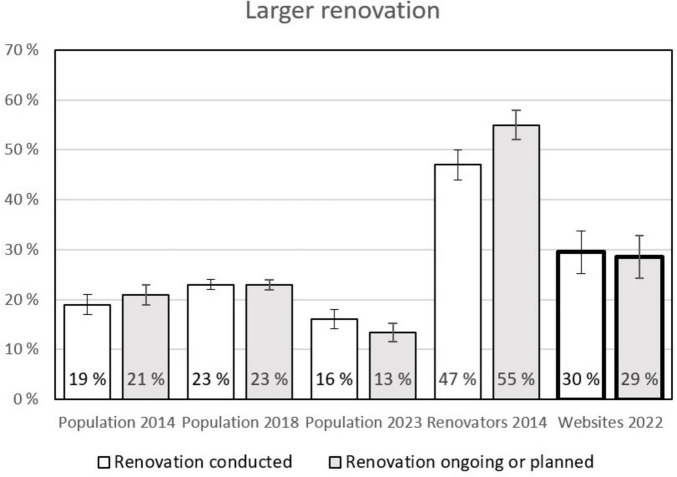
Percentage of households per group who were, are or plan to be in a deep renovation project (see definition in the text). The columns with the bold lines are the users of the counseling websites, whiskers represent 95% confidence intervals (CI), non-overlapping CI are regarded as indicating a statistically significant difference.

As can be seen in Figure 2, the percentage of people who were involved in a deep renovation project is higher in the group of counseling website users than in all three population samples. The same can be said for the ongoing or planned deep renovation projects, which are also more common for people visiting the energy counseling websites. Only the group that was specifically recruited in 2014 to only contain respondents who either just had been, were still, and/or were planning a deep renovation project in the near future has higher numbers (which is not surprising). Interestingly, the number of finished and planned projects in the population sample is lower in 2023 than in 2018 and 2014, likely an effect of renovation saturation after COVID years.

**FIGURE 3:**
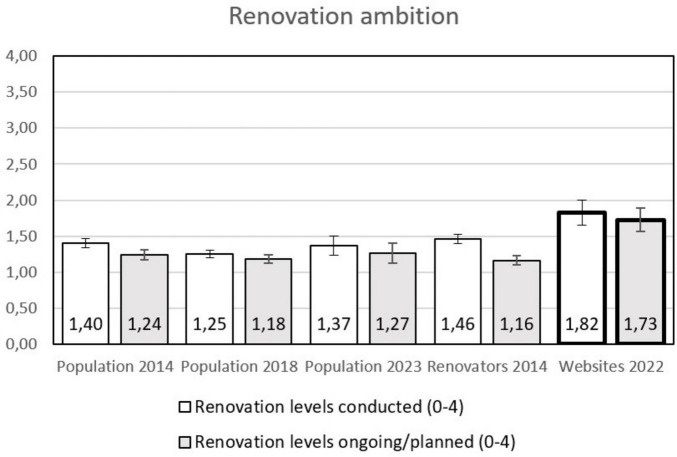
Ambition of the deep renovation (how many different measures are included of walls, windows, roof, and basement). The columns with the bold lines are the users of the counseling websites, whiskers represent 95% confidence intervals (CI), non-overlapping CI are regarded as indicating a statistically significant difference.

Among the users of the energy counseling websites, the ambition level is higher than in any other group, both for finished, ongoing and planned projects (see Figure 3). This means that they are engaged in slightly larger projects, involving more of the four different potential measures (walls, windows, roof, foundation). Thus, these people probably are or plan to be involved in more comprehensive renovation projects.

### 3.2 Energy efficiency ambitions

**FIGURE 4:**
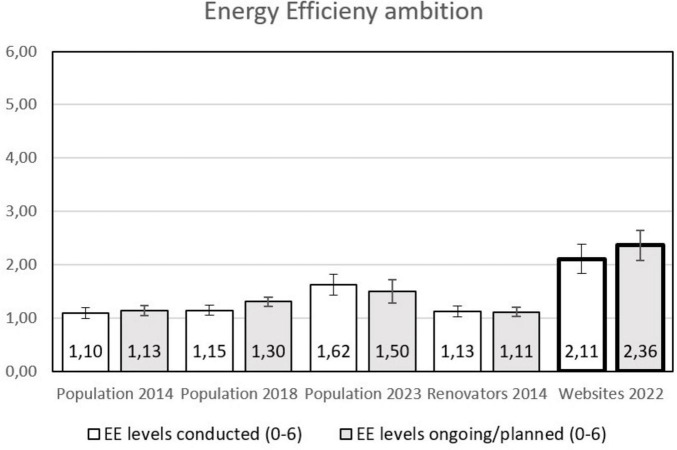
Ambition of the energy retrofit as part of the renovation (how many different energy efficiency measures are included of more insulation of walls, better windows, more insulation of roof and basement, balanced ventilation system, and heat pump). The columns with the bold lines are the users of the counseling websites, whiskers represent 95% confidence intervals (CI), non-overlapping CI are regarded as indicating a statistically significant difference.

When looking at the level of ambitions for integrating energy efficiency upgrades in the renovation projects, the picture is even more interesting (see Figure 4). Among the users of the energy counseling websites, the ambition level is substantially higher than in any other group, both for finished, ongoing, and planned projects. On a side note, even if the total percentage of people involved in deep renovation was lower in the population in 2023 than in 2014 and 2018, the degree to which energy efficiency measures are included is increasing as can be seen in Figures 2, 4. This may be an effect of the energy crisis in Europe in 2022.

### 3.3 Psychological drivers

**FIGURE 5:**
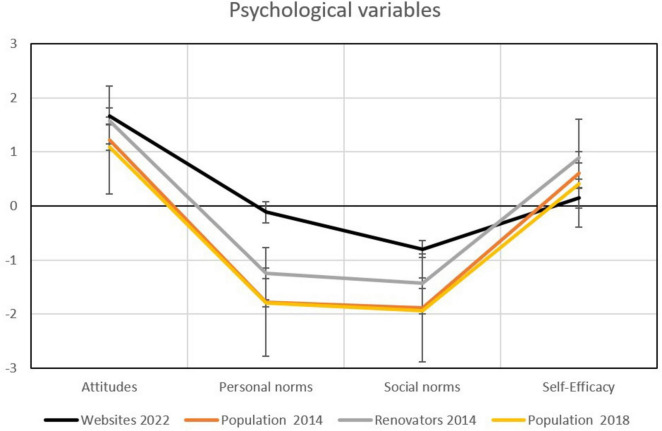
Means in key psychological variables driving the decision to renovate and energy upgrade. The bold black line is the sample from the counseling websites, whiskers represent 95% confidence intervals (CI), non-overlapping CI are regarded as indicating a statistically significant difference.

When comparing the psychological profiles of the website users to the population profiles from 2014 and 2018, it can be seen that the website users have substantially higher personal norms. This indicates that they feel more moral pressure to increase the energy efficiency of their dwellings (see Figure 5). They also feel stronger social norms, meaning more social pressure from their peers to engage in such energy upgrades. For attitudes, the differences are smaller. Meanwhile, the attitudes are slightly more positive than for the population samples, on the same level as for the renovators in 2014. Interestingly, despite small differences, the website users have the lowest perceived self-efficacy, especially compared to the renovators in 2014. In contrast to renovators in 2014, they feel less convinced that they know how to go about for the renovations.

### 3.4 Facilitators and barriers of energy efficiency upgrades

**FIGURE 6:**
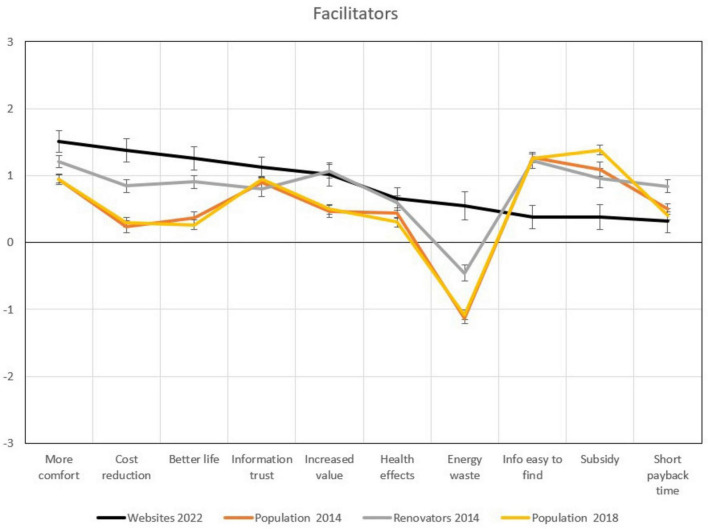
Means in key facilitators for an energy upgrade. The bold black line is the sample from the counseling websites, whiskers represent 95% confidence intervals (CI), non-overlapping CI are regarded as indicating a statistically significant difference.

**FIGURE 7:**
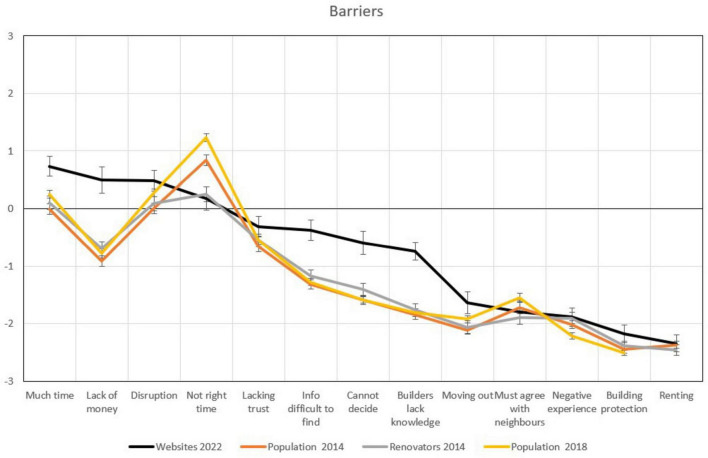
Means in key barriers towards an energy upgrade. The bold black line is the sample from the counseling websites, whiskers represent 95% confidence intervals (CI), non-overlapping CI are regarded as indicating a statistically significant difference.

Figures 6, 7 show how the website users perceive facilitators and barriers of energy efficiency upgrades of their dwellings in comparison to people in the other samples. For some facilitators and barriers, differences are substantial: counseling website users expect more comfort, a cost reduction, a house that is better to live in, increased property value, and less waste of energy as a result of the renovation. They score the lowest of all samples, though, on availability of information, payback time, and availability of subsidy.

For the barriers, they score particularly high on perceptions of the renovation taking too much time, on lack of money, difficulty of finding information, a lack of ability to decide what to do, and a lack of capable contractors. They score lower on perceptions of it not being the right time to act.

### 3.5 Implemented energy efficiency actions

In the one-year follow-up, the participants of the energy counseling website survey were contacted again and asked if they implemented the planned actions. 201 participants (46.0% of all participants) gave permission to be contacted a year after the initial survey was completed, and 78 (38.8% of all who were willing to be contacted) answered the short follow-up survey.

Of the 78 participants, 25 stated that they implemented the energy efficiency upgrades that they were planning to implement (32.1%). 29.2% of these changed at least 50% of the outer walls, 45.8% worked on the roof, 45.8% on the windows, and 37.5% on the foundation walls.

Of the 25 who implemented the measures, 15 added at least 5 cm insulation to the walls, 13 installed highly efficient windows (μ = 1.0 or smaller), 13 installed new mechanical ventilation, 12 insulated the roof with at least 10 cm additional insulation, 10 insulated the foundation walls with at least 5 additional cm of insulation, and 7 installed a balanced ventilation system. In addition to these measures, 11 installed heat pumps, 11 installed clean-burning wood stoves, and 5 installed solar panels on their houses. Overall, the measures taken were fairly ambitious.

The main reasons for not implementing the planned measures among the remaining participants of the follow-up were lack of economic funding (57.1%), lack of subsidies (42.9%), and that the time was not right, yet, to start the renovation, again reflecting some of the main barriers indicated in the introduction.

## 4 Discussion

The study conducted with the users of two energy efficiency counseling websites had three aims: (a) finding out if users of the website differed from representative samples of Norwegian households in terms of engagement in retrofits and have higher ambitions for their renovation projects and the energy efficiency measures embedded in them, (b) finding out if they differ in the psychological profile in central variables driving the decision-making process, and (c) finding out if they perceive facilitators and barriers in this process differently than representative samples of households. Furthermore, a follow-up study aimed to find out how many participants implement their ambitions up to a year later.

For all three main questions, we find substantial differences. Whereas the website users are mostly comparable to the general population of Norwegian households regarding socio-demographics (but have a higher education level and an even smaller percentage of people renting their dwelling, which reflects well the drivers for renovation projects as identified by Pardalis, 2021), their psychological profile differs in two important points. Compared to all other samples (also including the renovators studied in 2014), the website users have far higher levels of personal norms−they feel they really should do something about the energy standard of their homes−and also higher social norms. Considering the importance of these two factors for intentions to implement energy renovations (Klöckner and Nayum, 2017, 1014), this finding is relevant. Having such high levels of these two variables makes it more likely that people will form intentions to improve the energy standard of their homes. It also indicates that people like these are a prime target group for interventions like OSSs: They are already motivated to take action because they have high energy-related moral standards, and they feel the social pressure of their peer groups.

Since we could not survey these people before they went to the website, we do not know if they had such high personal and social norm values already before the visit to the website. On the other hand, since one of the websites is promoted by the environmental organization Friends of the Earth Norway, it can be assumed that this is the case. Interestingly, users of the counseling websites had a slightly lower level of self-efficacy, especially compared to the renovators from 2014. This implies that a lower level of self-efficacy might be a barrier to implement the intentions they form, and maybe also a reason for visiting the websites. Again, this means that this group is a very attractive target group for OSS-type interventions: Alleviating the low self-efficacy is something a well-designed OSS can achieve by reducing uncertainties, providing requested information, and not the least making the link between the urge to act on the side of the homeowners and the competence the homeowners are lacking provided by skilled and trustworthy contractors. This finding is, again, very much in line with what Pardalis (2021) found as being the most important features of OSSs from the perspective of potential users.

Also in terms of facilitators and barriers analysed, counseling website users had some values substantially different from the other groups. In particular, increased expected comfort levels, expected cost reductions, and expectations of having a better house to live in after the renovation were more important facilitators for website users than for the population samples or the renovators. Expecting an increased value of the house after the renovation was also higher than for the population samples, but at the same level as for the renovators. Perceiving the current energy standards a waste was standing out again for the website users. This indicates that they enter the process with a different, more energy interested perspective (or they get convinced of that by visiting the website). Interestingly, counseling website users score lower on perceptions that information is easy to find, and that access to subsidy is available. Maybe this is also a reason why they ended up on the websites in the first place.

Among the barriers, the website users mention a lot more often the time demand for supervision and the lack of money as the main barriers. They thereby raise the need to have a facilitator (or even a manager) of the renovation process, again a function OSSs typically fill. The websites we studied are following a facilitation model, but still leave the management of the project to the homeowners. From their answers, we can conclude that many of them would actually prefer a more comprehensive model. Also here, they reiterate that they consider information hard to find, that they cannot decide what to do, and that contractors lack competence. The latter three again might be reasons for being interested in the website services in the first place. The websites seem to partly satisfy their needs, as can be seen in that a significant amount of the website visitors implement their renovation plans within a year. However, some still sit with the same lack of support and the same barriers after a year. Maybe for them, a more comprehensive OSS model with a higher degree of process management would be more appropriate. In line with the renovators from 2014, the website users are to a lesser degree unsure if the right point in time for a renovation project has come. Overall, the order of importance of renovation facilitators and barriers to a large extent reproduces what has been found in earlier studies (Klöckner et al., 2013; Klöckner and Nayum, 2016, 2017; Bertoldi et al., 2021; Xue et al., 2022).

Most importantly, we found that the visitors of the websites had stronger ambitions for their renovation projects, and in particular for the implementation of energy efficiency measures as part of them. Of course, we do not know if this was caused by visiting the websites or if it was already higher before they visited. Nevertheless, we can assume that there is at least some mutual influence. People with a stronger motivation, but who are unsure about how to implement, visit the websites, which then confirm their motivations and provide hands-on counseling to remove the implementation barriers. This then eventually might result in higher ambitions. This is good news for the OSS concept, even the low-threshold version of it that these websites represent (McGinley et al., 2020). However, not all visitors seem to receive from these websites what they need. For the future, it might be recommendable to use low-threshold OSSs like the ones studied here following a facilitating model as an entry point but implement an (automated, maybe AI-based) detection of who would benefit from more comprehensive OSS models to channel these people to the offers that better suit their needs.

Finally, we could at least tentatively show−even if based upon only relatively few cases and subject to large sample attrition−that about 1/3 of the participants manage to implement their energy upgrade intentions. These people usually combine several measures and implement a deep renovation. For these people, the websites seem to have pushed them in the right direction without too much effort. As such, these websites have their niche as gatekeepers for a deeper process for some people, as the final push and reassurance for others.

## 5 Limitations and future research needs

Even if the study presented here shows some interesting results in a field where more research is needed, there are a number of limitations that are mostly caused by the design we had to choose. The biggest limitation of this study is that the participants recruited among the website users were, for obvious reasons, not randomly assigned to use the website but self-selected, and they were not surveyed before the visit on the website, a limitation that was already discussed in the methodology section. In addition, the users of the website fall into a narrower sociodemographic category than the population samples, though they seem to be rather comparable with people engaged in renovation projects six years prior to our study. Furthermore, we do not know how long people stayed on the websites, what they read, and how much they used the information to adapt their renovation strategy.

To address these limitations, studies with more controlled experimental designs would be advisable. Assigning participants randomly to different conditions (including no OSS, and different models of OSS) would give a better understanding of what the effects of the OSS are and what differences people come with in the process. Such a study could also test, whether different forms of OSS interact with different sociodemographic and psychological profiles of homeowners. In simple words, it might answer the question, which form of OSS works for which type of homeowner.

## 6 Conclusion

One-stop-shops have been promoted as a measure to overcome the inertia in energy efficiency retrofitting, especially in the privately owned residential building stock. Results from our study on users of two Norwegian energy efficiency counseling websites, which offer services in many ways similar to an OSS following a facilitator model, show that the users of these websites clearly differ from representative samples of Norwegian households that were surveyed with similar instruments. Their profiles were more like a sample of people who were in the beginning or in the middle of a larger renovation project, which was surveyed in 2014. However, the results also show that they are scoring substantially lower on their perceived access to information and subsidy. Regarding the psychological profiles, they were much more strongly motivated by personal and social norms than average households. Most importantly, it appears that visitors of such low-threshold websites have substantially higher ambitions for the energy upgrades, which about 1/3 of them have implemented a year after they visited the websites. Interest in online energy efficiency counseling services seems to be impacted by societal discussions about energy and/or by energy prices, as suggested by the spike in recruitment to our survey coinciding with an energy price increase during 2022 (however, this intriguing possibility will need to be confirmed in future studies). From a policy perspective, the results are interesting because they indicate that low-threshold OSSs can be gateways capturing people who are motivated for energy efficiency upgrades but not able to make the decision for several reasons. For some of them, the services that these relatively simple online platforms can offer is already enough to reduce their uncertainty and make the missing connections. For those still not satisfied after visiting these platforms, future developments should explore whether they can be automatically directed to more comprehensive forms of OSSs.

## Data availability statement

The datasets presented in this study can be found in online repositories. The names of the repository/repositories and accession number(s) can be found below: https://zenodo.org/records/10453810.

## Ethics statement

The studies involving humans were approved by the Norwegian Agency for Shared Services in Education and Research (SIKT). The studies were conducted in accordance with the local legislation and institutional requirements. The participants provided their written informed consent to participate in this study.

## Author contributions

CK: Conceptualization, Data curation, Formal analysis, Funding acquisition, Investigation, Methodology, Project administration, Supervision, Validation, Visualization, Writing–original draft, Writing–review and editing. AN: Data curation, Formal analysis, Writing–original draft, Writing–review and editing. SV: Conceptualization, Funding acquisition, Writing–original draft, Writing–review and editing.

## Footnotes

1
https://zenodo.org/records/12605729
