# Supplementary material for: Understanding users of online energy efficiency counseling: comparison to representative samples in Norway
Source: Front Psychol. 2024 Aug 6;15:1364980. doi: 10.3389/fpsyg.2024.1364980 (PMC11333433; doi:10.3389/fpsyg.2024.1364980)
Supplement: Supplementary file 1 [file Table_1.pdf]

Table A1

	Website -> 2014	Website -> 2018	Website -> 2023	Website -> Renovators
Renovation conducted	0,247	0,149	0,324	-0,365
Renovation ongoing/planned	0,177	0,128	0,382	-0,555
Renovation levels conducted	0,492	0,616	0,470	0,407
Renovation levels ongoing/planned	0,562	0,605	0,527	0,654
EE levels conducted	0,732	0,738	0,337	0,702
EE levels ongoing/planned	0,905	0,822	0,593	0,929
Attitudes	0,313	0,387	-	0,066
Personal norms	0,966	0,981	-	0,637
Social norms	0,715	0,755	-	0,392
Self-Efficacy	-0,242	-0,137	-	-0,418
More comfort	0,332	0,347	-	0,189
Cost reduction	0,624	0,601	-	0,316
Better life	0,474	0,543	-	0,207
Information trust	0,143	0,119	-	0,203
Increased value	0,293	0,284	-	-0,029
Health effects	0,121	0,205	-	0,036
Energy waste	0,833	0,817	-	0,496
Info easy to find	-0,519	-0,545	-	-0,498
Subsidy	-0,420	-0,625	-	-0,336
Short payback time	-0,096	-0,045	-	-0,305
Much time	0,416	0,281	-	0,358
Lack of money	0,639	0,574	-	0,549
Disruption	0,258	0,117	-	0,218
Not right time	-0,327	-0,546	-	-0,035
Lacking trust	0,195	0,137	-	0,133
Info difficult to find	0,551	0,538	-	0,464
Cannot decide	0,540	0,543	-	0,437
Builders lack knowledge	0,721	0,707	-	0,665
Moving out	0,270	0,155	-	0,239
Must agree with neighbours	-0,037	-0,114	-	0,044
Negative experience	0,090	0,228	-	0,012
Building protection	0,198	0,246	-	0,152
Renting	0,016	-	-	0,080
